# Reconstruction of metabolic pathway for isobutanol production in *Escherichia coli*

**DOI:** 10.1186/s12934-019-1171-4

**Published:** 2019-07-18

**Authors:** Shuhei Noda, Yutaro Mori, Sachiko Oyama, Akihiko Kondo, Michihiro Araki, Tomokazu Shirai

**Affiliations:** 1 Center for Sustainable Resource Science, RIKEN, 1-7-22, Suehiro-cho, Tsurumi-ku, Yokohama, Kanagawa 230-0045 Japan; 2 Department of Chemical Science and Engineering, Graduate School of Engineering, Kobe University, 1-1 Rokkodai, Nada, Kobe, 657-8501 Japan; 3 Graduate School of Medicine, Kyoto University, 54 Kawahara-cho, Syogoin, Sakyo-ku, Kyoto, 606-8507 Japan

**Keywords:** Entner–Doudoroff pathway, *Escherichia coli*, Glucose, Isobutanol, Microbial catalysis

## Abstract

**Background:**

The microbial production of useful fuels and chemicals has been widely studied. In several cases, glucose is used as the raw material, and almost all microbes adopt the Embden–Meyerhof (EM) pathway to degrade glucose into compounds of interest. Recently, the Entner–Doudoroff (ED) pathway has been gaining attention as an alternative strategy for microbial production.

**Results:**

In the present study, we attempted to apply the ED pathway for isobutanol production in *Escherichia coli* because of the complete redox balance involved. First, we generated ED pathway-dependent isobutanol-producing *E. coli*. Thereafter, the inactivation of the genes concerning organic acids as the byproducts was performed to improve the carbon flux to isobutanol from glucose. Finally, the expression of the genes concerning the ED pathway was modified.

**Conclusions:**

The optimized isobutanol-producing *E. coli* produced 15.0 g/L of isobutanol as the final titer, and the yield from glucose was 0.37 g/g (g-glucose/g-isobutanol).

**Electronic supplementary material:**

The online version of this article (10.1186/s12934-019-1171-4) contains supplementary material, which is available to authorized users.

## Background

The microbial production of valuable compounds, such as chemicals, fuels, and drugs, has been widely researched for the last few decades. Using genetically and metabolically engineered microorganisms as the host strain, these useful compounds were produced mainly from glucose as the raw material [1–6]. In nature, almost all microbes follow the Embden–Meyerhof (EM) pathway as the central metabolism system to acquire energy from carbohydrates such as glucose. In the EM pathway, 1 mol of glucose is metabolized to 2 mol of pyruvate via 10-step reactions. In the process, particularly bacteria can acquire 2 mol of adenosine triphosphate (ATP) and 2 mol of reduced nicotinamide adenine dinucleotide (NADH). *E. coli* and other bacteria follow an alternative metabolic pathway called as the Entner–Doudoroff (ED) pathway. In this pathway, similar to the EM pathway, 1 mol of glucose is metabolized to 2 mol of pyruvate; however, this pathway involved only 4 enzymes. Using the ED pathway, bacteria can acquire 1 mol of ATP, NADH, and reduced NADH phosphate (NADPH) [7].

Some reports concerning microbial production of valuable chemicals using the ED pathway are available. Ng et al. have introduced a synthetic ED pathway from *Zymomonas mobilis* into *E. coli* to produce terpenoid [8]. Zhao et al. have reported ethanol production from lignocellulosic biomass using *Z. mobilis* as the host strain, which originally adopted ED pathway to acquire energy from carbohydrates [9]. In various microorganisms, researches regarding applying the ED pathway for bioproduction have been widely undertaken [10–13].

**Fig. 1:**
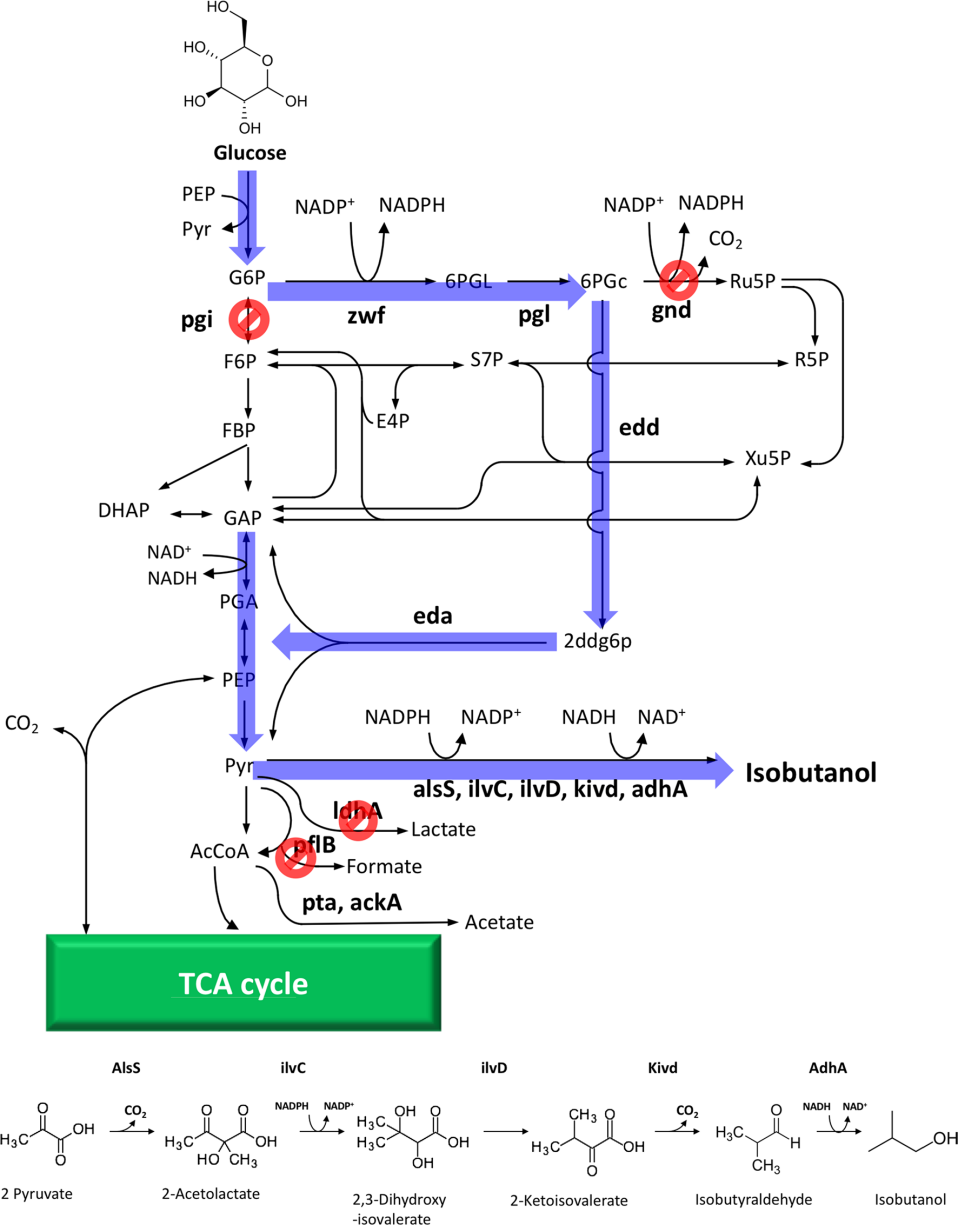
The synthetic metabolic pathway for isobutanol production in *E. coli.* PEP, phosphoenolpyruvate; AcCoA, acetyl coenzyme A; Pyr, pyruvate; NADPH, reduced nicotinamide adenine dinucleotide phosphate; NADP^+^, oxidised NADPH; NADH, reduced nicotinamide adenine dinucleotide; NAD^+^, oxidised NADH; G6P, glucose 6-phosphate; F6P, fructose 6-phosphate; FBP, fructose 1,6-bisphosphate; GAP, glyceraldehyde 3-phosphate; DHAP, dihydroxyacetone phosphate; PGA, glycerate 3-phosphate; 6PGL, 6-phospho-d-glucono-1,5-lactone; 6PGc, 6-phospho-d-gluconate; 2ddg6p, 2-dehydro-3-deoxy-d-gluconate 6-phosphate; Ru5P, d-ribulose 5-phosphate; R5P, d-ribose 5-phosphate; Xu5P, d-xylulose 5-phosphate; S7P, d-sedoheptulose 7-phosphate; E4P, d-erythrose 4-phosphate; pgi, glucose-6-phosphate isomerase; gntR, DNA-binding transcriptional repressor; gnd, 6-phosphogluconate dehydrogenase; pflB, pyruvate formate lyase; ldhA, d-lactate dehydrogenase; pta, phosphate acetyltransferase; zwf, glucose-6-phosphate dehydrogenase; pgl, 6-phosphogluconolactonase; edd, phosphogluconate dehydratase; eda, KHG/KDPG aldolase; ackA, acetate kinase; alsS, acetolactate synthase; ilvC, ketol-acid reductoisomerase; ilvD, dihydroxy-acid dehydratase; kivd, 2-ketoisovalerate decarboxylase; adhA, alcohol dehydrogenase

Isobutanol, one of the most attractive biofuels, has been widely studied for industrial production. A synthetic pathway of isobutanol has previously been constructed, and the suitable genes were isolated [14, 15]. This synthetic pathway initiates from the aldol condensation of 2 mol of pyruvate to 2-acetolactate by acetolactate synthase (AlsS) from *Bacillus subtilis*. In addition, the overexpression of the enzymes ketol-acid reductoisomerase (IlvC) and dihydroxy-acid dehydratase (IlvD) from *E. coli* and 2-ketoisovalerate decarboxylase (Kivd) and alcohol dehydrogenase (AdhA) from *Lactococcus lactis* contribute to the synthesis of isobutanol [14]. In this synthetic pathway, 1 mol of NADH and NADPH were consumed (Fig. 1). In the ED pathway of *E. coli*, 1 mol of NADH and NADPH were produced from 1 mol of glucose, as shown above. Therefore, the combination of ED pathway and the isobutanol synthetic pathway is expected to be one of the powerful strategies to achieve high isobutanol production because of the complete redox balance (Fig. 1). Although isobutanol has reportedly been produced using *E. coli* carrying a synthetic ED pathway of *Z. mobilis* [16], the EM pathway and pentose phosphate pathway (PPP) has remained in the metabolic pathway of *E. coli* used in the report; this indicates that the metabolic pathway for isobutanol production involving the ED pathway should be improved from the perspective of redox balance.

In the present study, we performed isobutanol production using genetically modified *E. coli* available for the ED pathway as the host strain. For this purpose, we generated *E. coli* strain strictly available for the ED pathway by inactivating the genes encoding glucose-6-phosphate isomerase (Pgi) and 6-phosphogluconate dehydrogenase (Gnd) passing to the EM pathway and PPP, respectively. Thereafter, the pathways to produce byproducts, such as organic acids, were interrupted from the metabolic pathway of the *E. coli* strain available for the ED pathway. Finally, the ED pathway was modified by overexpressing the genes concerning the endogenous ED pathway in *E. coli*. Using the *E. coli* transformant thus generated, we successfully demonstrated isobutanol production. The redox balance in the metabolic pathway is one of the most important factors, and the use of ED pathway would be a promising tool for microbial production.

## Results

### Creation of *E. coli* strain available for the ED pathway

To create isobutanol-producing *E. coli* with the complete redox balance, we attempted to modify the metabolic pathway of *E. coli* K-12 MG1655 (called as CFTi2 in the present study). First, we inactivated *pgi* to prevent the carbon flux to the EM pathway (CFTi3). To activate the ED pathway, *gntR*-encoding DNA-binding transcriptional repressor was interrupted. Although the CFTi4 strain thus generated was available for the ED pathway, the carbon flux to PPP pathway had already remained and the redox balance was unsuitable for isobutanol production. To overcome this difficulty, *gnd*-encoding 6-phosphogluconate dehydrogenase was removed from CFTi4 strain and the completely ED pathway-dependent *E. coli* was constructed (CFTi5) (Fig. 1). Here, trace experiments with [1-^13^C]glucose were performed to verify that the constructed strain metabolized glucose using the ED pathway (Additional file 1: Fig. S1). By analyzing mass isotopomer distributions of TBDMS-derivatized Ala, it was confirmed (Additional file 1: Table S2). The strains constructed in the present study are shown in Table 1.

**Table 1:** Strains, plasmids, and transformants used in the present study

Strains or plasmids	Genotype	Source or reference
Strains		
NovaBlue	*endA1 hsdR17* (rK12^−^mK12^+^) *supE44 thi*-*I gyrA96 relA1 lac* recA1/F’ [*proAB*^+^ *lacI*^q^ ZΔ*M15*::Tn*10* (Tet^r^)]; used for gene cloning	Novagen
ATCC 700926	MG1655	American Type Culture Collection
CFTi2	ATCC 700926	ATCC
CFTi3	CFTi2Δ*pgi*	This study
CFTi4	CFTi3Δ*gntR*	This study
CFTi5	CFTi4Δ*gnd*	This study
CFTi6	CFTi5Δ*pflB*	This study
CFTi9	CFTi6Δ*ldhA*	This study
CFTi10	CFTi7Δ*pta*	This study
CFTi2a	CFTi2 harboring pTka	This study
CFTi3a	CFTi3 harboring pTka	This study
CFTi4a	CFTi4 harboring pTka	This study
CFTi5a	CFTi5 harboring pTka	This study
CFTi6a	CFTi6 harboring pTka	This study
CFTi9a	*CFTi9 harboring pTka*	This study
CFTi10a	CFTi10 harboring pTka	This study
CFTi21	CFTi2a harboring p23SCD	This study
CFTi31	CFTi3a harboring p23SCD	This study
CFTi41	CFTi4a harboring p23SCD	This study
CFTi51	CFTi5a harboring p23SCD	This study
CFTi61	CFTi6a harboring p23SCD	This study
CFTi91	CFTi9a harboring p23SCD	This study
CFTi101	CFTi10a harboring p23SCD	This study
CFTi91co	CFTi91 harboring pSAK	This study
*CFTi91zp*	CFTi91 harboring pSzp	This study
CFTi91zpee	CFTi91 harboring pSzpee	This study
Plasmids		
pTrcHis B	P_trc_, pBR322 ori, Amp^r^	Life Technologies
pZE12MCS	P_LlacO1_, colE1 ori, Amp^r^	Expressys
pZA23MCS	P_AlacO1_, p15A ori, Km^r^	Expressys
pZA23trc	P_trc_, p15A ori, Km^r^	This study
pSAK	P_AlacO1_, SC101 ori, Cm^r^	[23]
pZka	pZE12MCS containing *kivd* and *adhA*from *Lactococcus lactis*	This study
pTka	pTrcHis B containing *kivd* and *adhA*from *Lactococcus lactis*	This study
p23S	pZA23trc containing *alsS* from *B. subtilis*	This study
p23SCD	pZA23trc containing *alsS* from *B. subtilis*, *ilvC* and *ilvD* from *Escherichia coli*	This study
pSp	pSAK containing *pgl* from *E. coli*	
pSzp	pSAK containing *zwf* and *pgl* from *E. coli*	This study
pSzpee	pSzp containing *edd* and *eda* from *E. coli*	This study

Amp, ampicillin; Km, kanamycin; Cm, chloramphenicolp

**Fig. 2:**
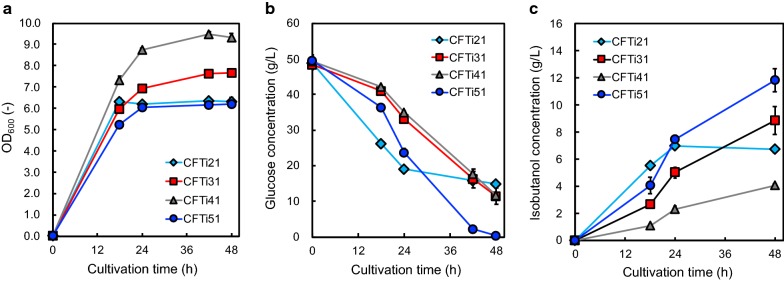
CFTi21, CFTi31, CFTi41, and CFTi51 culture profiles. Time courses of **a** bacterial cell growth and **b** glucose consumption. **c** The amount of isobutanol produced in CFTi21 (diamonds), CFTi31 (squares), CFTi41 (triangles), and CFTi51 (circles) cultures under aerobic condition. Data are presented as the mean ± standard deviation of three independent experiments

The synthetic gene clusters concerning isobutanol production were introduced into the *E. coli* strains generated above. Using these isobutanol-producing *E. coli* transformants, isobutanol production was evaluated under aerobic condition. Figure 2 shows the CFTi21, CFTi31, CFTi41, and CFTi51 culture profiles. The highest amount of isobutanol produced was 11.8 g/L in CFTi51 culture, which is a completely ED pathway-dependent strain, following 48-h cultivation, whereas CFTi51 is the unique strain that completely consumed glucose, its maximal cell growth was the lowest among the 4 transformants used in the present experiment. Although CFTi41 showed the highest cell growth, the amount of isobutanol produced in its culture was lowest among 4 strains. Moreover, we measured the amount of organic acids produced as byproducts. The ED pathway-dependent CFTi51 strain produced 1.5 g/L of acetate until 48-h cultivation, whereas the amount of the byproduct produced by CFTi21 that adopted the EM pathway was almost 3.0 g/L (Additional file 1: Fig. S2). The titer of isobutanol, yield from glucose, and rate of glucose uptake are summarized in Additional file 1: Table S3. The yield of isobutanol from glucose in CFTi51 after 48 h of cultivation was 0.24 g/g (g-isobutanol/g-glucose).

### Inactivation of the pathway to produce byproducts in *E. coli*

Furthermore, to prevent volatilization of isobutanol, test tubes used for cultivation were screw-capped after 18 h of cultivation. In this case, cultures were assumed to be relatively under anaerobic condition after 18 h of cultivation. In *E. coli* culture, under anaerobic condition, yielding organic acids, such as lactate, was possible. To further encourage isobutanol production, we attempted to disrupt the carbon flux to some organic aids as byproducts. Some organic acids, such as acetate, lactate, and formate are produced via pyruvate and acetyl coenzyme A (acetyl-CoA) in *E. coli*. Using CFTi5 as the base strain, *pflB*, *ldhA,* and *pta* (encoding pyruvate formate lyase, d-lactate dehydrogenase, and phosphate acetyltransferase, respectively) were sequentially inactivated (Fig. 1).

**Fig. 3:**
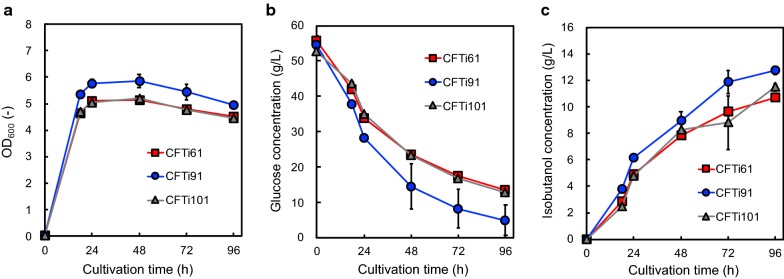
CFTi61, CFTi91, and CFTi101 culture profiles. The time courses of **a** bacterial cell growth and **b** glucose consumption. **c** The amount of isobutanol produced in CFTi61 (squares), CFTi91 (circles), and CFTi101 (triangles) cultures under aerobic (until 18 h) and anaerobic (after 18 h) conditions. Data are presented as mean ± standard deviation of three independent experiments

Using the modified isobutanol-producing *E. coli*, we performed isobutanol production. Figure 3 shows the CFTi61, CFTi91, and CFTi101 culture profiles. The maximal levels of cell growth, glucose consumption, and isobutanol production are shown in CFTi91 culture. Although inactivation of ldhA was effective in repressing lactate formation and improving isobutanol production, this production was decreased by removing the gene concerning acetate production (Fig. 3; Additional file 1: Fig. S3). The titer of isobutanol, yield from glucose, and rate of glucose uptake are summarized in Additional file 1: Table S3. The yield of isobutanol from glucose in CFTi91 between 18 and 96 h of cultivation was 0.28 g/g.

### Modification of ED pathway in isobutanol-producing *E. coli*

To further improve isobutanol production, we attempted to modify the ED pathway. Using a low copy number vector, the gene sets concerning the endogenous ED pathway in *E. coli* were introduced into isobutanol-producing *E. coli* transformant CFTi91 generated in the present study.

**Fig. 4:**
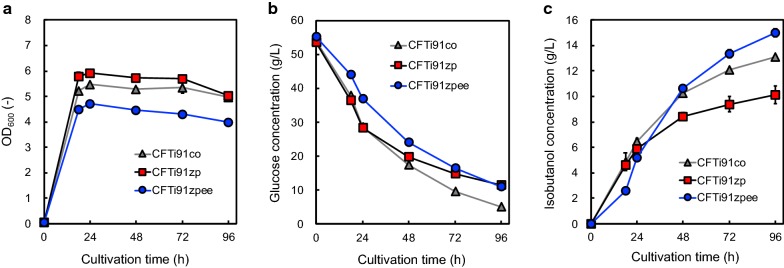
CFTi91co, CFTi91zp, and CFTi91zpee culture profiles. The time courses of **a** bacterial cell growth and **b** glucose consumption. **c** The amount of isobutanol produced in CFTi91co (triangles), CFTi91zp (squares), and CFTi91zpee (circles) cultures under aerobic (until 18 h) and anaerobic (after 18 h) conditions. Data are presented as mean ± standard deviation of three independent experiments

Figure 4 shows CFTi91co, CFTi91zp, and CFTi91zpee culture profiles. The highest amount of isobutanol produced was 15.0 g/L in CFTi91zpee culture, which carries the gene set of *zwf*, *pgl*, *edd,* and *eda* encoding glucose-6-phosphate dehydrogenase, 6-phosphogluconolactonase, phosphogluconate dehydratase, and KHG/KDPG aldolase, respectively. Although the maximal level of cell growth and the initial rates of glucose uptake and isobutanol production until 18-h cultivation in CFTi91zpee were lower than those in CFTi91co and CFTi91zp, this production was greater in CFTi91zpee than in the others after 18-h cultivation (Fig. 4). Further, we estimated the amount of organic acids produced as byproducts. Among the CFTi91-derivative strains used, acetate and succinate production were primarily confirmed. However, their total amount was < 1.0 g/L in each strain (Additional file 1: Fig. S4). The titer of isobutanol, yield from glucose, and rate of glucose uptake are summarized in Table 2. The yield of isobutanol from glucose in CFTi91zpee between 18 and 96 h cultivation was 0.37 g/g, and the final isobutanol titer was 15.0 g/L.

**Table 2:** Summary of isobutanol production by each engineered *E. coli* strain

Strain	P_max_ (g/L)	Yield (g/g)	Glucose uptake rate (g/L/h)	Production (g/L/h)
CFTi61	10.7 ± 0.1	0.28 ± 0.01	0.36 ± 0.01	0.10 ± 0.00
CFTi91	12.8 ± 0.3	0.28 ± 0.02	0.42 ± 0.05	0.12 ± 0.05
CFTi101	11.5 ± 0.3	0.29 ± 0.02	0.39 ± 0.01	0.11 ± 0.01
CFTi91co	13.1 ± 0.0	0.25 ± 0.01	0.42 ± 0.01	0.10 ± 0.01
CFTi91zp	10.1 ± 0.7	0.22 ± 0.01	0.32 ± 0.01	0.07 ± 0.01
CFTi91zpee	15.0 ± 0.4	0.37 ± 0.01	0.42 ± 0.00	0.16 ± 0.01

P_max_, the maximum amount of produced isobutanol

## Discussion

Recently, microbial production using various microbes has been widely studied toward industrial application. The tools of metabolic engineering and synthetic biology could be combined to design and create our desired microorganisms [17–19]. The metabolic pathway comprises several enzymatic reactions, particularly redox reactions. Hence, it is important to design the metabolic pathway toward the compounds of interest by considering the intracellular redox balance. We adopted the ED pathway to produce isobutanol using *E. coli*. In the ED pathway, each 1 mol of NADH and NADPH is produced from 1 mol of glucose, whereas each 1 mol of NADH and NADPH is consumed to produce 1 mol of isobutanol (Fig. 1) [7]. In the present study, we generated the ED pathway-dependent *E. coli* transformants and performed isobutanol production using the derivative strains.

Several studies regarding isobutanol production using various microbes have been reported. Atsumi et al. have reported isobutanol production using *E. coli* for the first time [15]. Moreover, *Corynebacterium glutamicum*, *B. subtilis*, and *Saccharomyces cerevisiae* were used for isobutanol production as the host strains; however, the maximal titer and yield levels have been obtained using *E. coli* [20–22]. In these reports, the inactivation of genes concerning byproducts formation, direct evolution of the enzymes in the biosynthetic pathway of isobutanol, and optimization of culture conditions were performed as the general strategies to improve isobutanol production. A study on isobutanol production has demonstrated the use of partially ED pathway-dependent *E. coli,* which adopted the ED pathway derived from *Z. mobilis* [16]. However, the strain used in that study was simply introduced to the genes concerning the ED pathway, and the redox balance in the biosynthesis pathway involved in converting glucose to isobutanol was not completely considered. The yield of isobutanol from glucose (0.31 g/g) was lower than that in the case using the EM pathway and has the potential for further improvements [16, 20].

In the present study, we constructed the ED pathway-dependent *E. coli* transformants by inactivating the negative repressor of the ED pathway and the genes concerning the initial reaction of EM and PPP pathway (Fig. 1). First, we inactivated *pgi* to repress the carbon flux to the EM pathway. The rates of glucose uptake and isobutanol production in CFTi31 were decreased, and acetate production as the byproduct was lower than that of CFTi21, which is completely dependent on the EM pathway (Additional file 1: Fig. S2). CFTi21 produced isobutanol mainly in growth phase, whereas did not in stationary phase (Fig. 2). This may be because CFTi21 produced a large amount of acetate along with isobutanol and decrease of pH caused lysis of bacterial cell. Although *gntR* was disrupted after removing *pgi* to activate the ED pathway, the amount of isobutanol production in CFTi41 was drastically decreased compared with that in CFTi21 or CFTi31 (Fig. 2). This could be attributed to the cell growth of CFTi41, which was greater than that of other strains used in this section; moreover, the PPP pathway and TCA cycle, which are important for cell growth may be activated in CFTi41, whose *pgi* and *gntR* were inactivated (Fig. 2). Finally, *gnd* catalyzing the third reaction of the PPP pathway was inactivated. Although the cell growth of CFTi51 was decreased compared with that of CFTi41, the isobutanol production was almost threefold higher (Fig. 2). This could be attributed to the repression of carbon leak to the PPP pathway and stimulation of the carbon flux to isobutanol from glucose through the ED pathway. The maximal titer and yield of isobutanol were recorded in the ED pathway-dependent CFTi51.

Thereafter, we changed the culture condition to prevent volatilization of isobutanol. Although culturing was initiated in aerobic condition, test tubes used here were screw-capped after 18 h of cultivation. To prevent the formation of organic acids associated with the change in culture condition, we inactivated genes concerning organic acid formation. Although the disruptions of *pflB* and *ldhA* concerning formate and lactate formation, respectively, were effective to improve isobutanol production, the inactivation of *pta* gene resulted in decreasing isobutanol production (Fig. 3). However, the amount of acetate produced in CFTi91 was only 250 mg/L, and the carbon from glucose would be used for isobutanol production (Additional file 1: Fig. S3). Here, we speculated the reason why the deletion of *pta* gene had a negative effect to isobutanol production, despite of lacking anaerobic source of acetyl-CoA which is a substrate of Pta. As shown in Additional file 1: Fig. S3A, the isobutanol-producing strain whose *pta* gene was deleted drastically decreased acetate production. We assumed that a small amount of produced acetate until 24 h cultivation (aerobic or micro-aerobic condition) was one of key factor in the isobutanol production using our created strain. The energy balance optimized by deleting both pflB and ldhA genes might be disturbed by omitting the gene concerning acetate production.

To further improve isobutanol production, we attempted to modify the endogenous ED pathway in *E. coli*. Although the initial isobutanol production and the maximal cell growth were decreased by introducing 4 genes constructing the ED pathway, the final titer of isobutanol and yield from glucose were encouraged (Fig. 4; Table 2). In the optimized strain CFTi91zpee as well as in CFTi91, acetate production was repressed, whereas succinate production was relatively increased (Additional file 1: Fig. S4). In the present study, the maximal yield of isobutanol from glucose reached 0.37 g/g, which was higher than that in a previous report using partially ED pathway-dependent strain for isobutanol production and 90% of the maximal theoretical yield (0.41 g/g) [16]. In addition, it would be comparable with other reports on isobutanol production using various host strains (Table 3) [20]. These results indicate that the redox-balanced metabolic design is one of the significant factors in microbial production. Although we reconstructed metabolic pathway suitable for isobutanol production from the aspect of the redox balance, *E. coli* is known to have the endogenous transhydrogenase encoded in *pntAB* concerning the conversion of NADH into NADPH which is a candidate reaction to acquire NADPH [21]. In the previous report about isobutanol production, the coexpression of PntAB and NAD kinase contributed to encouraging isobutanol production [22]. This result would mean that those two enzymes needed to be overexpressed to earn the enough activity of converting NADH to NADPH, which is one of essential cofactors for isobutanol biosynthetic pathway.

**Table 3:** Summary of microbial production of isobutanol

Host	Genotype (knockout)	P_max_ (g/L)	Yield (g/g)	References
*E. coli* MG1655	Δ*pgi*Δ*gntR*Δ*gnd* Δ*pflB*Δ*ldhA* (Available for an endogenous ED pathway)	15.0	0.37	This study
*E. coli* MG1655	Δ*pflB*Δ*ldhA*Δ*ackA*-*pta* (Carrying an exogenous ED pathway)	13.7	0.31	[16]
*E. coli* BW25113	Δ*adhE*Δ*ldhA*Δ*frdBC* Δ*fnr*Δ*pta*Δ*pflB* (EM pathway)	22	0.35	[15]
*C. glutamicum* ATCC13032	Δ*aceE*Δ*pqo*Δ*ilvE*Δ*ldhA* Δ*mdh* (EM pathway)	13	0.2	[23]
*B. subtilis*	Δ*ldhA* (EM pathway)	2.62	N.E.	[24]

NE, not estimated

In the experiment keeping under anaerobic condition after 18 h cultivation, succinate production was confirmed (Additional file 1: Figs. S3C and S4B). the isobutanol-producing strain used in the study lacked the intracellular CO_2_ sources the gene *pflB*, oxidative TCA cycle, and the oxidative pentose phosphate pathway, however; 2 mol of CO_2_ was produced in the exogenous biosynthetic pathway of isobutanol (Fig. 1). Succinate production would be attributed to the activation of phosphoenolpyruvate (PEP) carboxylase which produces oxaloacetate from PEP and CO_2_. The produced oxaloacetate might be converted into succinate via the reductive TCA cycle to prevent overflowing the intracellular excess energy.

In conclusion, for effective isobutanol production, we generated ED pathway-dependent *E. coli* strain, which produced 11.8 g/L of isobutanol, and the yield from glucose was 0.24 g/g under aerobic condition. By inactivating genes concerning organic acids and modifying the endogenous ED pathway, isobutanol production was increased, and the titer and yield from glucose were 15.0 g/L and 0.37 g/g, respectively. The optimization of culture conditions and the collection of volatilized isobutanol are expected to further improve isobutanol production.

## Methods

### Strains and plasmid construction

The strains and plasmids used in the present study are listed in Table 1 and Additional file 1: Table S1. *E. coli* NovaBlue competent cells (Novagen, Cambridge, MA, USA) were used for gene cloning. Polymerase chain reaction (PCR) was performed using the KOD FX Neo DNA polymerase (Toyobo, Osaka, Japan), and the primer pairs are listed in Additional file 1: Table S1. Each gene was assembled with the respective plasmid using Gibson Assembly (New England Biolabs, Ipswich, MA, USA). The construction of plasmids used in this study is described in Table 1. The plasmids were transformed into bacterial strains using the Gene Pulser II (Bio-Rad, Hercules, CA, USA). Wherever applicable, 100 µg/mL ampicillin, 50 µg/mL kanamycin, and 15 µg/mL chloramphenicol were added to the media for selection purpose. All the transformants constructed in this study are listed in Table 1.

pZA23trc was constructed as follows: The nucleotide sequence of *trc* promoter and pZA23 without the promoter region were amplified by PCR using pTrcHis B and pZA23MCS as the templates with the primer pairs inv_pZA23_no_prom_f and inv_pZA23_no_prom_r. The amplified fragments were fused with each other, and the resulting plasmid was designated as pZA23trc. pZka and pTka were constructed as follows: the synthetic gene fragments of *kivd* and *adhA* were directly cloned into the *Kpn*I and *Hin*dIII sites of pZE12MCS, respectively, and the resulting plasmid was designated as pZka. Subsequently, the gene set of *kivd* and *adhA* and the plasmid fragment of pTrcHis B were amplified by PCR using pZka and pTrcHis B as the template with the primer pair kivd_ptrc_f and adhA_ptrc_r and inv_ptrc_f and inv_ptrc_r, respectively. The amplified fragments were fused with each other, and the resulting plasmid was designated as pTka. p23S and p23SCD were constructed as follows: *alsS* (NP_391482.2) and pZA23trc fragment were amplified by PCR using *B. subtilis* genomic DNA and pZA23trc as the template with the primer pairs alsS_ptrc_f and alsS_ptrc_r and inv_ptrc_f and inv_ptrc_r, respectively. The amplified fragments were fused together, and the resulting plasmid was designated as p23S. Thereafter, the gene set of ilvC (NP_418222.1) and ilvD (YP_026248.1) was amplified by PCR using *E. coli* K-12 MG1655 genomic DNA as the template and the primer pair ilvCD_ptrc_sacI _f and ilvCD_ptrc_sacI_r. The amplified fragment was cloned into the *Sac*I site of p23S, and the resulting plasmid was designated as p23SCD. pSp, pSzp, and pSzpee were constructed as follows: *pgl* (NP_415288.1) and pSAK fragment were amplified by PCR using *E. coli* K-12 MG1655 genomic DNA and pSAK as the template and the primer pairs pgl_pZ_hindIII_f and pgl_pZ_hindIII_r and inv_pZ_hindIII_f and inv_pZ_hindIII_r, respectively. The amplified fragments were fused together, and the resulting plasmid was designated as pSp. *zwf* (NP_416366.1) was amplified by PCR using *E. coli* K-12 MG1655 genomic DNA as the template with the primer pair zwf_pZ_kpnI _f and zwf_pZ_kpnI _r. The amplified fragment was cloned into the *Kpn*I site of pSp, and the resulting plasmid was designated as pSzp. *edd* (NP_416365.1) and *eda* (NP_416364.1) were amplified by PCR using *E. coli* K-12 MG1655 genomic DNA as the template with the primer pairs edd_pZ_bamHI _f and edd _r and eda_f and eda_pZ_bamHI_r, respectively. The gene set of *edd* and *eda* were amplified by PCR using these amplified fragments of *edd* and *eda* as the templates with the primer pair i edd_pZ_bamHI _f and eda_pZ_bamHI_r. The amplified fragment was cloned into the *Bam*HI site of pSzp, and the resulting plasmid was designated as pSzpee. Plasmid maps used in this study are shown in Additional file 1: Fig. S5.

### Deletion of chromosomal genes

The primers used for the deletion of chromosomal genes are listed in Additional file 1: Table S1. *E. coli* K-12 MG1655 (ATCC700926) was used as the parent strain. Using the Quick and Easy *E. coli* Gene Deletion Kit (Funakoshi, Tokyo, Japan), each gene was deleted according to the manufacturer’s protocol. The gene fragments for deletion and recombination were introduced into each strain using the Gene Pulser II. Other fragments used to inactivate the respective genes were amplified by PCR using the FRT-PGK-gp2-neo-FRT template DNA from the Quick and Easy *E. coli* Gene Deletion Kit with the appropriate primers, as listed in Additional file 1: Table S1.

### Culture conditions

The M9Y medium was used for isobutanol production in 5-mL test tube-scale cultures. M9Y minimal medium contains (per liter): glucose, 20 g; yeast extract, 5 g; NaCl, 0.5 g; Na_2_HPO_4_‧12H_2_O, 17.1 g; KH_2_PO_4_, 3 g; NH_4_Cl, 2 g; MgSO_4_‧7H_2_O, 246 mg; CaCl_2_‧2H_2_O, 14.7 mg; FeSO_4_‧7H_2_O, 2.78 mg; thiamine hydrochloride, 10 mg. Wherever applicable, ampicillin, kanamycin, and/or chloramphenicol were added to the medium at a final concentration of 100, 50, and 15 µg/mL, respectively. Each preculture was seeded to 5 mL of M9Y medium in a test tube at an initial OD_600_ of 0.05. Test tube-scale cultures were incubated at 37 °C in a shaker at 220 rpm. IPTG (0.1 mM) was added to the culture medium at OD_600_ of 0.5, following which test tube-scale cultures were incubated at 30 °C in a shaker at 220 rpm. The trace experiments with [1-^13^C]glucose were performed as below. The M9 medium was used in 5-mL test tube-scale cultures. M9Y minimal medium contains (per liter): [1-^13^C]glucose, 20 g; NaCl, 0.5 g; Na_2_HPO_4_‧12H_2_O, 17.1 g; KH_2_PO_4_, 3 g; NH_4_Cl, 2 g; MgSO_4_‧7H_2_O, 246 mg; CaCl_2_‧2H_2_O, 14.7 mg; FeSO_4_‧7H_2_O, 2.78 mg; thiamine hydrochloride, 10 mg. Other culture conditions were the same as isobutanol-production experiments.

### Analytical methods

Cell growth was monitored by measuring OD_600_ with a UVmini-1240 spectrophotometer (Shimadzu, Kyoto, Japan). The concentration of glucose in the culture supernatant was measured using the Glucose CII test (Wako, Kyoto, Japan) according to the manufacturer’s protocol.

Gas chromatography–mass spectrometry (GC-MS) was performed using a GCMS-QP2010 Ultra instrument (Shimadzu) equipped with a DB-FFAP column (60 m, 0.25-mm internal diameter, 0.5-mm film thickness; Agilent). Helium was used as the carrier gas to maintain a flow rate of 2.1 mL/min. The injection volume was 1 μL with a split ratio of 1:10. The amount of isobutanol was quantified as follows: the oven temperature was initially maintained at 40 °C for 1 min, following which it was gradually raised to 195 °C at 10 °C/min and further to 250 °C at 120 °C/min before finally being maintained at 250 °C for 3 min. The total running time was 20 min. The other settings included maintaining the interface temperature at 250 °C, ion-source temperature at 200 °C, and electron impact ionization at 70 eV.

Further, the concentration of organic acids was determined in the culture supernatants that were separated from the culture broth by centrifugation at 21,880×*g* for 20 min. The concentrations of organic acids as byproducts were determined using an organic acid analysis system (Shimadzu) consisting of an HPLC instrument equipped with a Shim-pack SPR-H column. The column was operated at 48 °C with a flow rate of 0.8 mL min. CDD-10A was used as the detector. *p*-Toluene sulphonic acid (5 mM) was used as the mobile phase, and 20-mM bis–Tris containing 5-mM *p*-toluene sulphonic acid and 100-μM ethylenediaminetetraacetic acid was mixed immediately before the detection to enhance the sensitivity.

## Additional file

**Additional file 1.** Additional files and tables.

## Data Availability

The datasets supporting this work are included in the manuscript and additional file.

## Abbreviations

EM pathway: Embden–Meyerhof pathway
ED pathway: Entner–Doudoroff pathway
ATP: adenosine triphosphate
NADH: reduced nicotinamide adenine dinucleotide
NADPH: reduced nicotinamide adenine dinucleotide phosphate
PPP: pentose phosphate pathway
PCR: polymerase chain reaction
GC–MS: gas chromatography–mass spectrometry
acetyl-CoA: acetyl coenzyme A
