# Supplementary material for: Reconstruction of metabolic pathway for isobutanol production in Escherichia coli
Source: Microb Cell Fact. 2019 Jul 18;18:124. doi: 10.1186/s12934-019-1171-4 (PMC6637570; doi:10.1186/s12934-019-1171-4)
Supplement: Supplementary file 1 — Additional file 1. Additional files and tables. [file 12934_2019_1171_MOESM1_ESM.docx]

**Additional Information**

Reconstruction of metabolic pathway for isobutanol production in *E. coli*

Shuhei Noda^1^, Yutaro Mori^1^, Sachiko Oyama^1^, Akihiko Kondo^2^, Michihiro Araki^3^ & Tomokazu Shirai ^1^*

^1^ Center for Sustainable Resource Science, RIKEN, 1-7-22, Suehiro-cho, Tsurumi-ku, Yokohama, Kanagawa 230-0045, Japan.

^2^ Department of Chemical Science and Engineering, Graduate School of Engineering, Kobe University, 1-1 Rokkodai, Nada, Kobe 657-8501, Japan.

^3^ Graduate School of Medicine, Kyoto University, 54 Kawahara-cho, Syogoin, Sakyo-ku, Kyoto 606-8507, Japan.

**Correspondence:** [Tomokazu Shirai, Center for Sustainable Resource Science, RIKEN, 1-7-22, Suehiro-cho, Tsurumi-ku, Yokohama, Kanagawa 230-0045, Japan].

E-mail: [tomokazu.shirai@riken.jp](mailto:tomokazu.shirai@riken.jp)

**Table S1. Oligonucleotide primers used in the present study**

| Oligonucleotide primers | Sequence |
| --- | --- |
| ptrc_to_pZA23_f | 5′-CGTCTTCACCTCGAGTGTTGACAATTAATCATCCG-3′ |
| ptrc_to_pZA23_r | 5′-GAATATATCCCTAGGAAGGCCCAGTCTTTCGACTG-3′ |
| inv_pZA23_no_prom_f | 5′-CCTAGGGATATATTCCGCTT-3′ |
| inv_pZA23_no_prom_r | 5′-CTCGAGGTGAAGACGAAAGG-3′ |
| kivd_ptrc_f | 5′-CATCATCATCATGGTATGTATACCGTGGGTGATTA-3′ |
| adhA_ptrc_r | 5′-TCTCGAGCTCGGATCTTATTTGGTAAAATCGATCA-3′ |
| inv_ptrc_f | 5′-GATCCGAGCTCGAGATCTGCAGCT-3′ |
| inv_ptrc_r | 5′-ACCATGATGATGATGATGAGAACC-3′ |
| alsS_ptrc_f | 5′-CATCATCATCATGGTATGTTGACAAAAGCAACAAA-3′ |
| alsS_ptrc_r | 5′-TCTCGAGCTCGGATCCTAGAGAGCTTTCGTTTTCA-3′ |
| ilvCD_ptrc_sacI_f | 5′-ATAAGGATCCGAGCTTTAAGAGTTATGGCTAACTACTTCAATAC-3′ |
| ilvCD_ptrc_sacI_r | 5′-TGCAGATCTCGAGCTTTAACCCCCCAGTTTCGATT-3′ |
| pgl_pZ_hindIII_f | 5′-TCGACGGTATCGATAAGAGGAGAAAATGAAGCAAACAGTTTATAT-3′ |
| pgl_pZ_hindIII_r | 5′-AGGAATTCGATATCATTAGTGTGCGTTAACCACCA-3′ |
| inv_pZ_hindIII_f | 5′-TGATATCGAATTCCTGCAGC-3′ |
| inv_pZ_hindIII_r | 5′-TATCGATACCGTCGACCTCG-3′ |
| zwf_pZ_kpnI_f | 5′-AGAGGAGAAAGGTACATGGCGGTAACGCAAACAGC-3′ |
| zwf_pZ_kpnI_r | 5′-GGGGGGGCCCGGTACTTACTCAAACTCATTCCAGG-3′ |
| edd_pZ_bamHI_f | 5′-TCCTGCAGCCCGGGGAAGAGGAGAAAGGTACATGAATCCACAATTGTTACG-3′ |
| edd_r | 5′-TTAAAAAGTGATACAGGTTG-3′ |
| eda_f | 5′-TGTATCACTTTTTAAAAGAGGAGAAAGGTACATGAAAAACTGGAAAACAAG-3′ |
| eda_pZ_bamHI_r | 5′-GCACGCGTACCATGGTTACAGCTTAGCGCCTTCTA-3′ |
| delta_pgi_f | 5′-ATGAAAAACATCAATCCAACGCAGACCGCTGCCTGGCAGGCACTACAGAAAATTAACCCTCACTAAAGGGCG-3′ |
| delta_pgi_r | 5′-TTAACCGCGCCACGCTTTATAGCGGTTAATCAGACCATTGGTCGAGCTATTAATACGACTCACTATAGGGCTC-3′ |
| delta_gntR_f | 5′-ATGAAAAAGAAAAGACCCGTACTTCAGGATGTGGCTGACCGTGTAGGCGTAATTAACCCTCACTAAAGGGCG-3′ |
| delta_gntR_r | 5′-TTAAATAGATCCGCCCGGTGACAAGGTGAAACCTAAATCTAACATTTTCGTAATACGACTCACTATAGGGCTC-3′ |
| delta_gnd_f | 5′-ATGTCCAAGCAACAGATCGGCGTAGTCGGTATGGCAGTGATGGGACGCAAAATTAACCCTCACTAAAGGGCG-3′ |
| delta_gnd_r | 5′-TTAATCCAGCCATTCGGTATGGAACACACCTTCTTTATCAATACGCTTATTAATACGACTCACTATAGGGCTC-3′ |
| delta_pflB_f | 5′-ATGTCCGAGCTTAATGAAAAGTTAGCCACAGCCTGGGAAGGTTTTACCAAAATTAACCCTCACTAAAGGGCG-3′ |
| delta_pflB_r | 5′-TTACATAGATTGAGTGAAGGTACGAGTAATAACGTCCTGCTGCTGTTCTTTAATACGACTCACTATAGGGCTC-3′ |
| delta_ldhA_f | 5′-ATGAAACTCGCCGTTTATAGCACAAAACAGTACGACAAGAAGTACCTGCAAATTAACCCTCACTAAAGGGCG-3′ |
| delta_ldhA_r | 5′-TTAAACCAGTTCGTTCGGGCAGGTTTCGCCTTTTTCCAGATTGCTTAAGTTAATACGACTCACTATAGGGCTC-3′ |
| delta_pta_f | 5′-GTGTCCCGTATTATTATGCTGATCCCTACCGGAACCAGCGTCGGTCTGACAATTAACCCTCACTAAAGGGCG-3′ |
| delta_pta_r | 5′-TTACTGCTGCTGTGCAGACTGAATCGCAGTCAGCGCGATGGTGTAGACGATAATACGACTCACTATAGGGCTC-3′ |

**Table S2. Mass isotopomer data of TBDMS-derivatized alanine obtained by removing natural isotope abundances from the raw GC­–MS data[2].**

|  | Mass isotopomer | Value | STDEV | NatAb |
| --- | --- | --- | --- | --- |
| AlaM-57 | m0  m1  m2  m3 | 0.659  0.335  0.007  0.000 | 0.002  0.003  0.001  0.000 | 0.967  0.032  0.000  0.000 |
| AlaM-85 | m0  m1  m2 | 0.975  0.024  0.002 | 0.002  0.002  0.001 | 0.978  0.022  0.000 |

The values and STDEVs were obtained by four parallel experiments. Abbreviations: STDEV, standard deviation of mass isotopomer value; NatAb, theoretical value of natural abundance.

**Table S3. Summary of isobutanol production in the process in constructing ED pathway-dependent *E. coli***

| Strain | P_max_, time (g/L) | Yield (g/g) | Glucose uptake rate (g/L/h) |
| --- | --- | --- | --- |
|  |  |  |  |
|  |  |  |  |
| CFTi21 | 7.00 ± 0.29 (24 h) | 0.20 ± 0.01 | 0.71 ± 0.00 |
|  |  |  |  |
| CFTi31 | 8.86 ± 1.06 (48 h) | 0.24 ± 0.01 | 0.77 ± 0.07 |
|  |  |  |  |
| CFTi41 | 4.08 ± 0.08 (48 h) | 0.11 ± 0.01 | 0.78 ± 0.04 |
|  |  |  |  |
| CFTi51 | 11.83 ± 0.84 (48 h) | 0.24 ± 0.02 | 1.02 ± 0.03 |
|  |  |  |  |

P_max_, the maximum amount of produced isobutanol.

**Figure S1. Possibilities in labeling patterns of GC–MS data of Ala derived from pyruvate in case of metabolizing glucose via glycolysis, Entner–Doudoroff pathway, and pentose phosphate pathway.**


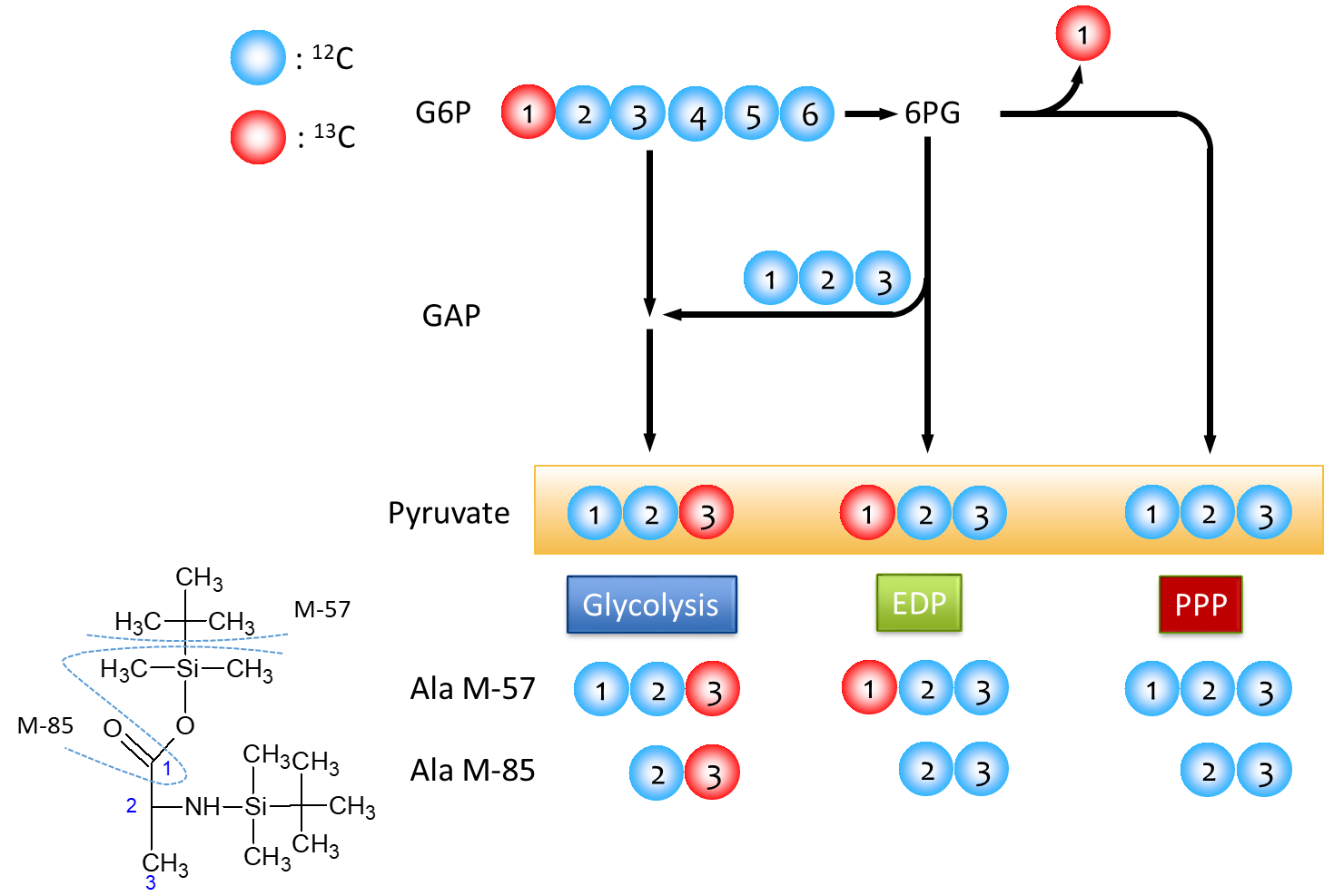


[1-^13^C]glucose is converted to 50 % of [3-^13^C]pyruvate via glycolysis, 50 %[1-^13^C]pyruvate via Entner–Doudoroff pathway, and natural abundance of ^13^C pyruvate via pentose phosphate pathway. Compared between two kinds of GC–MS data of TBDMS derivatized Ala M-57 and M-85, it is used to evaluate how the pyruvate produced is metabolized via Entner–Doudoroff pathway. Mass isotopomer distribution of Ala M-85 is the same as natural abundance of Ala M-85, even though Ala M-57 has ^13^C isotopomers, when glucose is metabolized only via Entner–Doudoroff pathway (See Table S1). Abreviations: G6P, glucose-6-phosphate; 6PG, 6-phosphogluconate; GAP, glyceraldehyde-3-phosphate; EDP, Entner-Doudoroff pathway; PPP, pentose phosphate pathway

**Figure S2 | Time courses of produced organic acids in CFTi21, CFTi31, CFTi41, and CFTi51 cultures**

Time courses of produced (A) acetate, (B) pyruvate, (C) succinate, and (D) malate in CFTi21 (diamonds), CFTi31 (squares), CFTi41 (triangles), and CFTi51 (circles) cultures. Data are presented as mean ± standard deviation of three independent experiments.

**Figure S3 | Time courses of produced organic acids in CFTi61, CFTi91, and CFTi101 cultures**

Time courses of produced (A) acetate, (B) lactate, and (C) succinate in CFTi61 (squares), CFTi91 (circles), and CFTi101 (triangles) cultures. Data are presented as the mean ± standard deviation of three independent experiments.

**Figure S4 | Time courses of produced organic acids in CFTi91co, CFTi91zp, and CFTi101zpee cultures**

Time courses of produced (A) acetate and (B) succinate in CFTi91co (triangles), CFTi91zp (squares), and CFTi91zpee (circles) cultures. Data are presented as mean ± standard deviation of three independent experiments.

**Figure S5 | Plasmid maps used in the present study**


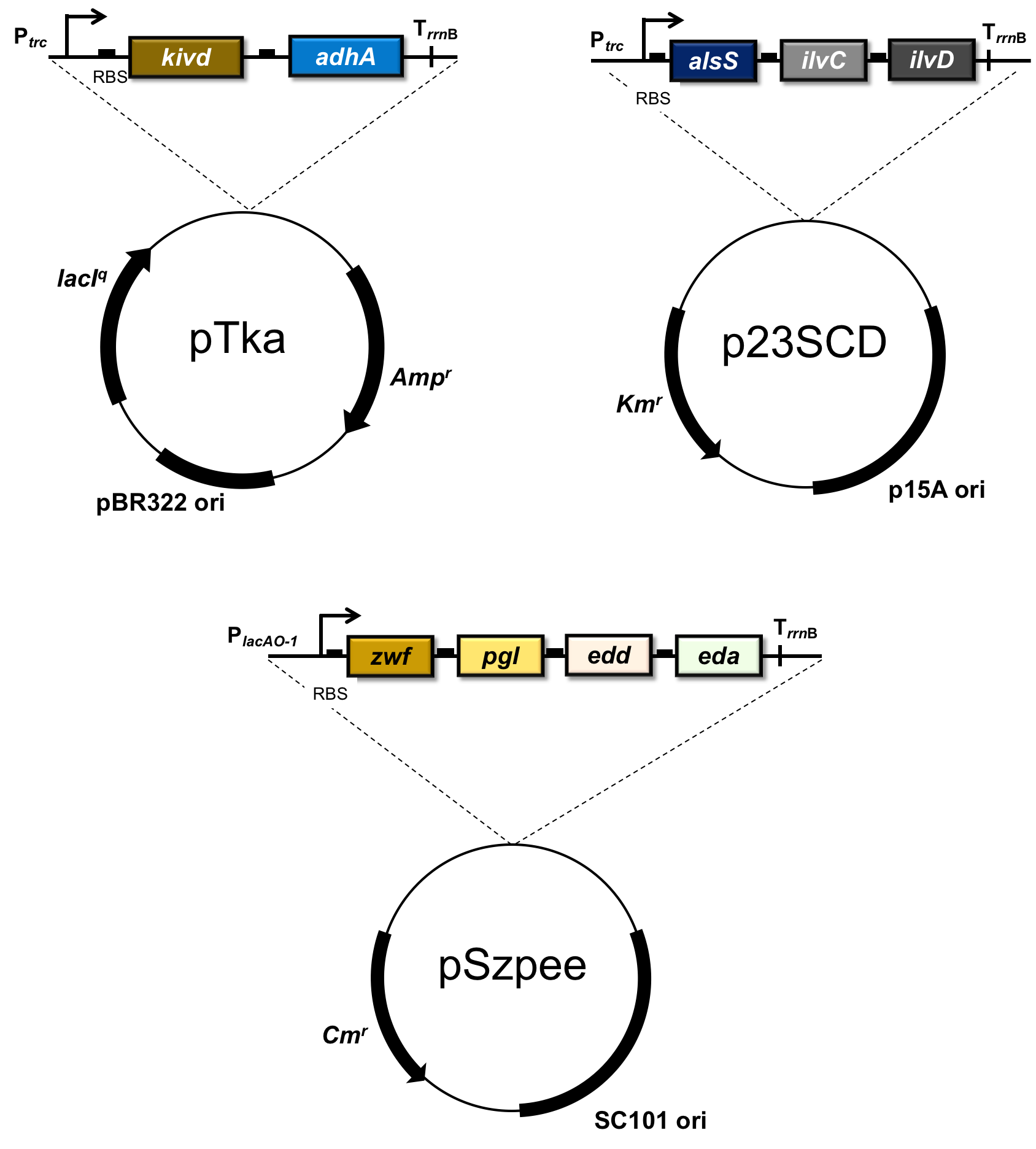


**Additional Materials**

**Sequence of the synthetic *kivd*:**

ATTAAAGAGGAGAAAGGTACCATGTATACCGTGGGTGATTATCTGCTGGATCGTCTGCATGAACTGGGTATTGAAGAAATTTTTGGTGTTCCGGGTGATTACAATCTGCAGTTTCTGGATCAGATTATCAGCCGCAAAGATATGAAATGGGTTGGCAATGCAAATGAACTGAATGCAAGCTATATGGCAGATGGTTATGCACGTACCAAAAAAGCAGCAGCATTTCTGACCACCTTTGGTGTTGGTGAACTGAGCGCAGTTAATGGTCTGGCAGGTAGCTATGCAGAAAATCTGCCGGTTGTTGAAATTGTTGGTAGCCCGACCAGCAAAGTTCAGAATGAAGGTAAATTTGTGCATCATACCCTGGCCGATGGTGATTTTAAACACTTTATGAAAATGCACGAACCGGTTACCGCAGCACGTACCCTGCTGACCGCAGAAAATGCAACCGTGGAAATTGATCGTGTTCTGAGCGCACTGCTGAAAGAACGTAAACCGGTGTATATTGATCTGCCTGTTGATGTTGCAGCAGCAAAAGCAGAAAAACCGCTGCTGCCGCTGAAAAAAGAAAATCCGACCAGTAATACCAGCGATCAAGAAATCCTGAACAAAATCCAAGAGAGCCTGAAAAACGCCAAAAAACCGATTGTTATTACCGGTCATGAAATCATTAGCTTCGGTCTGGAAAATACCGTGACCCAGTTTATTAGCAAAACCAAACTGCCGATTACCACCCTGAATTTTGGTAAAAGCAGCGTTGATGAAGCACTGCCGAGCTTTCTGGGTATCTATAATGGTAAACTGAGTGAGCCGAACCTGAAAGAATTTGTTGAAAGCGCAGACTTTATTCTGATGCTGGGTGTTAAACTGACCGATAGCAGTACCGGTGCATTTACCCATCATCTGAATGAGAACAAAATGATCAGCCTGAACATCGATGAAGGCAAAATCTTTAATGAACGCATCCAGAACTTTGACTTCGAAAGCCTGATTAGCAGCCTGCTGGACCTGAGCGAAATTGAATATAAAGGCGATTATATCGACAAAAAACAAGAAAACTTTGTGCCGAGCAATGCCCTGCTGAGCCAGGATCGCCTGTGGCAGGCAGTTGAAAGTCTGACCCAGAGCAATGAAACCATTGTTGCAGAACAGGGCACCAGTTTTTTTGGTGCAAGCAGTATTTTTCTGAAACCGAAAAGCCATTTTATTGGTCAGCCGCTGTGGGGTAGCATTGGTTATACCTTTCCGGCAGCACTGGGTAGCCAGATTGCAGATAAAGAAAGCCGTCATCTGCTGTTTATTGGTGATGGTAGCCTGCAGCTGACCGTTCAAGAACTGGGTCTGGCAATTCGTGAAAAAATCAATCCGATCTGCTTTATCATCAACAACGATGGCTATACCGTTGAACGTGAAATTCATGGTCCGAATCAGAGCTATAATGATATTCCGATGTGGAACTATTCGAAACTGCCGGAATCATTTGGTGCCACCGAAGATCGTGTTGTTAGCAAAATTGTTCGCACCGAAAATGAATTTGTGAGCGTTATGAAAGAAGCACAGGCCGATCCGAATCGTATGTATTGGATTGAACTGATTCTGGCCAAAGAAGATGCACCGAAAGTTCTGAAAAAAATGGGTAAACTGTTTGCCGAACAGAATAAAAGCTAAGGGCCCCCCCTCGAG

**Sequence of the synthetic *adhA*:** CGGTATCGATAAGCTAAAGAGGAGAAATGAAAGCAGCAGTTGTTCGTCATAATCCGGATGGTTATGCAGATCTGGTTGAAAAAGAACTGCGTGCCATTAAACCGAATGAAGCACTGCTGGATATGGAATATTGTGGTGTTTGTCATACCGATCTGCATGTTGCAGCCGGTGATTATGGTAATAAAGCAGGCACCGTTCTGGGTCATGAAGGTATTGGTATTGTTAAAGAAATTGGCACCGATGTTAGCAGCCTGCAGGTTGGTGATCGTGTTAGCGTTGCATGGTTTTTTGAAGGTTGTGGTCATTGCGAATATTGCGTTAGCGGTAATGAAACCTTTTGCCGTGAAGTTAAAAATGCCGGTTATAGCGTGGATGGTGGTATGGCAGAAGAAGCAATTGTTGTTGCAGATTATGCAGTTAAAGTGCCGGATGGTCTGGATCCGATTGAAGCAAGCAGCATTACCTGTGCCGGTGTTACCACCTATAAAGCAATTAAAGTTAGCGGTGTTAAACCGGGTGATTGGCAGGTTATTTTTGGTGCGGGTGGTCTGGGTAATCTGGCAATTCAGTATGCCAAAAATGTGTTTGGTGCCAAAGTTATCGCCGTGGATATTAATCAGGATAAACTGAACCTGGCCAAAAAAATCGGTGCCGATGTTATTATCAATAGCGGTGATGTTAATCCGGTGGATGAAATCAAAAAAATCACCGGTGGCCTGGGTGCACAGAGCGCAATTGTTTGTGCAGTTGCACGTATTGCATTTGAACAGGCAGTTGCAAGCCTGAAACCGATGGGTAAAATGGTTGCAGTTGCCCTGCCGAATACCGAAATGACCCTGAGCGTTCCGACCGTTGTTTTTGATGGTGTTGAAGTTGCAGGTAGCCTGGTTGGCACCCGTCTGGATCTGGCCGAAGCATTTCAGTTTGGTGCAGAAGGTAAAGTTAAACCGATTGTTGCAACCCGTAAACTGGAAGAAATTAACGATATCATCGACGAGATGAAAGCCGGTAAAATTGAAGGTCGTATGGTGATCGATTTTACCAAATAAAGCTTGATATCGAAT

**References**

[1] Noda S, Shirai T, Mori Y, Oyama S. et al. Engineering a synthetic pathway for maleate in *Escherichia coli*. Nat Commun*.* 2017;8:1153-1165.

[1] van Winden WA, Wittmann C, Heinzle E, Heijnen JJ, Correcting mass isotopomer distributions for naturally occurring isotopes. Biotechnol Bioeng. 2002;80:477-479.
